# History of current non-insulin medications for diabetes mellitus

**DOI:** 10.3402/jchimp.v2i3.19081

**Published:** 2012-10-15

**Authors:** Celeste C. L. Quianzon, Issam E. Cheikh

**Affiliations:** Division of Endocrinology, Department of Medicine, Union Memorial Hospital, Baltimore, MD, USA

**Keywords:** diabetes medication, history, review

## Abstract

This article is a brief review of the current non-insulin agents for diabetes mellitus in the United States, namely, sulfonylureas, biguanides, thiazolidinediones, meglitinides, α-glucosidase inhibitors, glucacon-like peptide-1 receptor agonists, dipeptidyl-peptidase-4 inhibitors, amylin agonists, bromocriptine, and colesevelam.

Since the introduction of sulfonylureas, multiple medications have been introduced for the treatment of diabetes mellitus type 2, substituting or supplementing insulin. A short review of these medications is presented in this article.

## Sulfonylureas

Sulfonylureas stimulate pancreatic β-cells to secrete insulin by binding to receptors that block the potassium ATP-dependent channels, leading to cell depolarization and subsequently insulin exocytosis. The hypoglycemic activity of synthetic sulfur compounds was noted by Ruiz and his colleagues in 1937 (1). In 1942, Janbon, a French physician, and his colleagues confirmed hypoglycemia in patients treated with *p*-amino-sulfonamide-isopropyl-thiodiazole for typhoid (1), and in August 1946, Lobatieres and his colleagues established that this group of drugs stimulated β-cell release of insulin (1). In 1956, the first sulfonylurea, tolbutamide, was introduced commercially in Germany followed by chlorpropamide, acetohexamide, and tolazamide, the first-generation sulfonylureas (1, 2). In 1984, more than 14 years after their introduction in Europe, glyburide and glipizide, which are more potent second-generation sulfonylureas, became available in the United States (3–6). Glimepiride, a third-generation sulfonylurea, was introduced in 1995 in the United States (7). The HbA1C (A1C) is decreased by 1–2%. Sulfonylureas have been in the market for more than 50 years. They are safe, cheap, and predictable, but the incidence of hypoglycemia, a major side effect, limits their use.

## Biguanides

The use of biguanide can be traced back to the medieval times when Galega officinalis, an herb, was used to relieve symptoms of diabetes (8). The plant was found to contain guanadine, a compound with hypoglycemic properties but too toxic for clinical use (9). Two synthetic diguanides were used between 1920 and 1930 but were discontinued from clinical use because of their toxic nature (8). In the 1950s, three biguanides, metformin, phenformin, and buformin, were introduced. Metformin and phenformin were introduced in the United States but were withdrawn in 1978 because use of phenformin led to increased incidences of lactic acidosis (8). In 1995, Metformin, which inhibits gluconeogenesis and improves peripheral glucose utilization, was reapproved in the United States after being in use in Europe for 20 years (10).

In 1998, the U K Prospective Diabetes Study (UKPDS)-34 examined the effect of intensive glucose control in overweight (mean BMI, 31), type 2 diabetes patients treated with metformin (11). UKPD study showed that metformin decreased the risk of diabetes-related end points and was associated with less weight gain and lesser hypoglycemic events compared with sulfonylureas and insulin (11).

Currently, metformin has been used for the first-line treatment of type 2 diabetes, alone or in combination with other diabetes agents, in addition to lifestyle modifications (12). A1C is decreased by 1–2%. An important contraindication for patients treated with biguanides is renal impairment, with creatinine level greater than 1.4 mg/dL and 1.5mg/dL for women and men, respectively. Lactic acidosis, the major side effect, is rarely observed when metformin is administered properly (13). Gastrointestinal side effects, such as nausea, diarrhea, and abdominal discomfort, may occur.

## Thiazolidinediones

Thiazolidinediones improve insulin sensitivity by binding to the peroxisome proliferator activator receptors in the target cell nucleus, which causes conformational changes with the retinoic X receptor. The discovery of thiazolidinediones was the result of the observation that patients with type 2 diabetes on clofibrate had lower fasting glucose levels (14). In the quest for formulating more potent fibrates in the early 1980s, Takeda Pharmaceuticals, Japan, made analogs of clofibrates that had positive effects on hyperglycemia, hyperinsulinemia, and hypertriglyceridemia in animals with type 2 diabetes. This led to the discovery of the first thiazolidinedione, ciglitazone, which had a modest effect on glucose and significant effects on lipids but caused edema and lenticular opacities in rodents (14). Ciglitazone was never marketed. In 1997, troglitazone became the first thiazolidinedione to be approved for clinical use. Though effective, it was withdrawn in 2000 after it was found to cause liver damage. Two other thiazolidinediones, rosiglitazone and pioglitazone, were approved in 1999 for treatment of type 2 diabetes. In September 2010, the US Food and Drug Administration (US FDA) restricted the use of rosiglitazone because of its potential to cause cardiovascular ischemia (15), and a recent study found that long-term use of pioglitazone slightly increases the risk of bladder cancer (16, 17).

The use of pioglitazone, alone or in combination with other diabetes agents, is permitted in the United States. A1C is decreased by 1–1.5%. The most common side effect is edema, which is dose related. Pioglitazone should be used with caution in patients with congestive heart failure (CHF) stage I and II, and it is contraindicated in CHF stage III and IV. Anemia and osteoporosis may also occur.

## Meglitinides

Meglitinides are non-sulfonylurea insulin secretagogues with short half-lives. These medications bind to the SUR1 binding site in the pancreas. They are given 15–30 min premeal to target the postprandial rise in glucose. Repaglinide is the first agent in this class to be approved for use in 1997 (18) followed by nateglinide in 2000 (19). A1C is decreased by 1–1.5%. The need for multiple meal-timed doses and the incidence of hypoglycemia limit their use.

## α-Glucosidase Inhibitors

α-Glucosidase inhibitors reversibly inhibit α-glucosidase enzyme present at the brush border membrane of the small intestine, which delays carbohydrate degradation and absorption, and thereby the subsequent desired effect of reduced postprandial hyperglycemia. Acarbose was approved by the US FDA in 1995 (20) and miglitol in 1996 (21). The effect on A1C is modest; it decreases by 0.5%. The need for multiple prandial dosing and gastrointestinal side effects of flatus and diarrhea markedly limit their use.

## Glucagon-Like Peptide-1 Receptor Agonists and Dipeptidyl Peptidase-4 Inhibitors

Glucagon-like peptide-1 (GLP-1) is a hormone secreted by the L cells of the small intestines within minutes following a carbohydrate- or fat-containing meal (22, 23). GLP-1 stimulates insulin synthesis and glucose-dependent insulin secretion. Moreover, GLP-1 suppresses glucagon release and delays gastric emptying (22, 23). It has a short half-life of 1–2 min because of rapid degradation by dipeptidyl peptidase-4 (DPP-4) (23). These physiologic benefits led to the development of GLP-1 receptor agonists and DPP-4 inhibitors for the treatment of type 2 diabetes (24).

Exenatide, the first GLP-1 agonist, is a mimetic of exendin-4, a peptide isolated from the saliva of the Gila monster, and has a 53% likeness to the human GLP-1 (25, 26). It is more resistant to DPP-4 degradation, thus has a longer half-life (26). Exenatide injection, twice daily, given 40–60 min before breakfast and dinner was approved in 2005 (27), and a once-weekly formulation was approved in January 2012 (28). Once-daily injection of liraglutide, a modified form of human GLP-1, with 97% homology, was approved in 2010 (29). GLP-1 receptor agonists decreases A1C by 1%. The most common side effects are gastrointestinal disorders, including nausea and occassional vomiting. Pancreatitis has also been reported (30, 31). There is concern for medullary thyroid cancer as it was seen in rats, though not reported in humans.

There are three approved DPP-4 inhibitors: sitagliptin, saxagliptin, and linagliptin. They effect a modest A1C reduction of up to 0.8%. They are available alone or in combination with metformin. The near absence of hypoglycemia makes their use desirable. It should be noted that pancreatitis has been reported among patients using DPP-4 inhibitors (32–34).

## Amylin Agonists

Amylin, a neuroendocrine hormone, was discovered in 1987. It is co-secreted with insulin by the pancreatic β-cells in a molar ratio (35, 36). Patients with type 1 diabetes have no amylin, whereas those with type 2 diabetes have a relative deficiency (36, 37). The physiologic effects of amylin include reduction of postprandial glucagon secretion and hepatic glucose production, resulting in lowering postprandial glucose levels. It also delays gastric emptying and mediates satiety (35–37). As amylin is insoluble and toxic to pancreatic β-cells, the development of a soluble amylin agonist facilitated a new class of diabetes medication. Pramlintide, a synthetic analog of amylin, was approved by the US FDA in 2005 as an adjunct to preprandial insulin therapy (38). The effect on A1C is a reduction by 0.4–0.6%. Pramlintide reduces prandial glucose, decreases prandial glucagon, delays gastric emptying, and induces weight loss (35). The most common side effect is nausea.

## Bromocriptine

Bromocriptine is an old drug with a new indication. It is a sympatholytic D2-receptor agonist approved in 2009 for the treatment of type 2 diabetes as an adjunct to diet and exercise (39). Oral administration of bromocriptine once daily within 2 hours of awakening reduced postprandial glucose levels (40). Its mechanism has not been fully understood, but it is thought to increase dopamine activity in the brain and inhibit excess sympathetic tone (40). A1C is decreased by up to 0.7%.

## Colesevelam

Colesevelam is a bile-acid sequestrant that reduces LDL cholesterol and lowers glucose levels (41). It is given twice daily. The mechanism of action is not known. Colesevelam was approved by the US FDA in 2008 for treatment of type 2 diabetes (42). The reduction in hemoglobin A1C is a modest 0.5%.

## Conclusion

Home glucose monitoring, education, and lifestyle changes enhance the management of all patients with diabetes mellitus. Advances in research, that is, diabetes gene therapy and human insulin-producing cell therapy, may personalize treatment and make cure possible.
